# Dr. Geetha Rangan

**Published:** 2010

**Authors:** Chandra P. Satish

**Affiliations:** Department of Neurology, NIMHANS, Bangalore, India. E-mail: iancon2006@gmail.com

**Figure:**
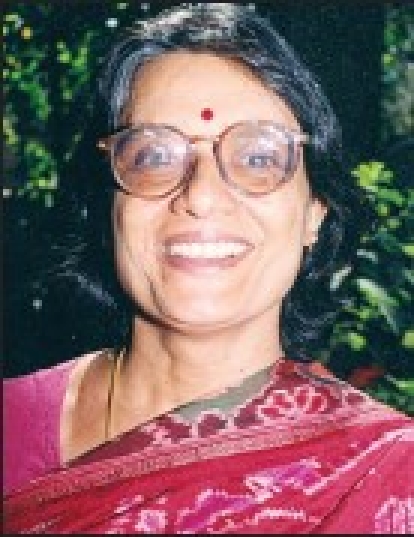
Geetha Rangan

Dr. Geeta Rangan a distinguished neurologist and epileptologist from Bangalore died prematurely on 16 March 2010 after ailing for nearly 4 months.

Geeta was born as a second child for Mr. K. S. Rangan and Ms. Lalitha Rangan on 3 February 1948. Her initial education was in Allahabad before joining Calcutta Medical College. She finished her medical degree with distinction, later moved to Delhi along with her parents as her father was transferred to New Delhi as Director, Department of Civil Aviation. She was selected for doing her MD (General Medicine) at AIIMS. She was one of the brightest postgraduate acclaimed for her research on Tuberculosis meningitis and passed out in 1973 with flying colors. Then she moved to Bangalore to pursue her DM (Neurology). She was hailed as very hard working, sincere, committed postgraduate senior resident in Neurology. During this time working under Professor K. S. Mani she developed keen interest in epilepsy. She was the fourth DM student from NIMHANS and first lady postgraduate in Neurology.

Subsequently, she married Professor K. S. Mani and together they established Neurology Clinic in private practice in Bangalore. She took greater role in carrying out famous "Yelandur Epidemiological Research Study." She was known for her meticulous record keeping and for outspoken nature.

She worked as a faculty and developed Department of Neurology in St. John's Medical College, Manipal Hospital and Sathya Sai Medical Institution at Bangalore. Again at the end of her career she developed another neurology department at Vydehi Medical College.

She was closely associated with Bangalore chapter of Indian Epilepsy Association for nearly two decades. She was editor of the IEA "News letter" for nearly a decade. She played very keen role in creating awareness of epilepsy in the community through theatre plays, children picnics etc. She has number of publications in the field of epilepsy along with Professor K. S. Mani.

She was a loner after the demise of Professor Mani, she spent her free time in gardening and playing with her pet dog Grabby.

At the time of her demise from this world, she was survived by her elderly mother aged 89 years and her elder brother Mr. Chandramouli.

Throughout her life, she was known for her bold, outspoken, straightforward nature. She will be remembered for her contribution to the Indian Epilepsy Association.

